# Supplementary material for: Effects of five types of exercise on vascular function in postmenopausal women: a network meta-analysis and systematic review of 32 randomized controlled trials
Source: PeerJ. 2024 Jul 15;12:e17621. doi: 10.7717/peerj.17621 (PMC11257064; doi:10.7717/peerj.17621)
Supplement: Supplemental Information 2 [file peerj-12-17621-s002.docx]

The rationale for conducting the systematic review / meta-analysis:

As women age, particularly after entering menopause, the prevalence of cardiovascular diseases (CVD) increases significantly. This makes it a critical global public health concern. Regular exercise can prevent and improve blood pressure and lipid levels in postmenopausal women. However, the most effective form of exercise to enhance vascular structure and function in this population remains uncertain. The objective of this study is to compare and analyze the effects of five different forms of exercise on vascular function in postmenopausal women. The study aims to determine the most effective exercise interventions to identify which modes of exercise training can maximally reduce cardiovascular risk in this population.

The contribution that it makes to knowledge in light of previously published related reports, including other meta-analyses and systematic reviews

The study has several strengths. Firstly, it utilized five types of exercise modalities that are easily accessible, widely applicable, and cost-effective. This provides motivation and convenience for postmenopausal women to engage in physical activity. Secondly, this study is the first to use NMA to assess the impact of various exercise modalities on five vascular function indicators in postmenopausal women. The study assesses the impact on vascular health by examining vascular structure, arterial endothelial function, arterial stiffness, and endothelial function. This analysis provides scientific evidence for exercise prescriptions and cardiovascular health management in postmenopausal women, helping to develop personalized and effective exercise regimens.
