# Supplementary material for: Effects of five types of exercise on vascular function in postmenopausal women: a network meta-analysis and systematic review of 32 randomized controlled trials
Source: PeerJ. 2024 Jul 15;12:e17621. doi: 10.7717/peerj.17621 (PMC11257064; doi:10.7717/peerj.17621)
Supplement: Supplemental Information 3 [file peerj-12-17621-s003.docx]

| **Supplemental materials** | **Page** |
| --- | --- |
| **Appendix 1.** Search strategy of PubMed, Embase, Cochrane, Web of Science,  and EBSCO. | 5-6 |
| **Appendix 2.** Characteristics of the included studies. | 7-15 |
| **Appendix 3.** List of included studies. | 16-17 |
| **Appendix 4.** Risk of bias assessment. | 18 |
| **Appendix 5.** Contributions of direct and indirect comparisons to NMA and the number of studies of each direct comparison of flow mediated dilation (FMD), pulse wave velocity (PWV), augmentation index (AIx), intima-media thickness (IMT), nitric oxide (NO). | 19 |
| **Appendix 6.** Inconsistency of vascular function outcomes tested by loop-specific heterogeneity estimates, inconsistency model, and node splitting analysis. | 20-21 |
| **Appendix 7.** Forest plots of eligible comparisons of flow mediated dilation (FMD), pulse wave velocity (PWV), augmentation index (AIx), intima-media thickness (IMT), nitric oxide (NO). | 22 |
| **Appendix 8.** The funnel plot graphics of flow mediated dilation (FMD), pulse wave velocity (PWV), augmentation index (AIx), intima-media thickness (IMT), nitric oxide (NO) in NMA. | 23 |
| **Appendix 9.** Area under the curve for cumulative ranking probability of  each intervention on flow mediated dilation (FMD), pulse wave velocity (PWV), augmentation index (AIx), intima-media thickness (IMT), nitric oxide (NO). | 23 |

**Appendix** **1.** Search strategy of PubMed, Embase, Cochrane, Web of Science, and EBSCO.

| **Database** | **Search Terms** | |  |  |
| --- | --- | --- | --- | --- |
|  | **Exercise** | **Postmenopause** | **vascular function** | **Randomized controlled**  **trial** |
| PubMed [Title/Abstract] | Exercise [MeSH Terms] OR sports OR training OR aerobic training OR aerobic exercise OR moderate intensity continuous training OR resistance training OR resistance exercise OR strength training OR strength exercise OR combined training OR combined exercise OR concurrent training OR high intensity interval training OR sprint interval training OR endurance training | Postmenopause [MeSH Terms] OR Post-Menopause* OR Post-menopausal Period OR Period, Post-menopausal OR Post Menopause*; Period OR Postmenopausal OR Postmenopausal Period OR old female OR elderly female OR elderly women | Vascular function OR vascular compliance OR vascular reactivity OR vascular stiffness OR vascular endothelium OR vasodilation OR endothelial function OR endothelial dysfunction OR arterial stiffness OR arterial distensibility OR pulse wave velocity OR macrovascular OR microvascular OR coronary flow reserve OR carotid intima-media thickness OR central blood pressure OR flow-mediated dilatation OR beta-stiffness index OR FMD OR PWV | Randomized controlled trial OR randomized OR placebo |
| Embase [Title/Abstract] | Training [Emtree term] or sports OR aerobic training OR aerobic exercise OR moderate intensity continuous training OR resistance training OR resistance exercise OR strength training OR strength exercise OR combined training OR combined exercise OR concurrent training OR high intensity interval training OR sprint interval training OR endurance training | Postmenopause [Emtree term] OR Post-Menopause* OR Post-menopausal Period OR Period, Post-menopausal OR Post Menopause*; Period OR Postmenopausal OR Postmenopausal Period OR old female OR elderly female OR elderly women | Vascular function OR vascular compliance OR vascular reactivity OR vascular stiffness OR vascular endothelium OR vasodilation OR endothelial function OR endothelial dysfunction OR arterial stiffness OR arterial distensibility OR pulse wave velocity OR macrovascular OR microvascular OR coronary flow reserve OR carotid intima-media thickness OR central blood pressure OR flow-mediated dilatation OR beta-stiffness index OR FMD OR PWV | Randomized controlled trial OR randomized OR placebo |
| Cochrane [Title/Abstract/ keywords] | Exercise [MeSH Terms] OR sports OR training OR aerobic training OR aerobic exercise OR moderate intensity continuous training OR resistance training OR resistance exercise OR strength training OR strength exercise OR combined training OR combined exercise OR concurrent training OR high intensity interval training OR sprint interval training OR endurance training | Postmenopause [MeSH Terms] OR Post-Menopause* OR Post-menopausal Period OR Period, Post-menopausal OR Post Menopause*; Period OR Postmenopausal OR Postmenopausal Period OR old female OR elderly female OR elderly women | Vascular function OR vascular compliance OR vascular reactivity OR vascular stiffness OR vascular endothelium OR vasodilation OR endothelial function OR endothelial dysfunction OR arterial stiffness OR arterial distensibility OR pulse wave velocity OR macrovascular OR microvascular OR coronary flow reserve OR carotid intima-media thickness OR central blood pressure OR flow-mediated dilatation OR beta-stiffness index OR FMD OR PWV | — |
| Web of Science | Exercise OR sports OR training OR aerobic training OR aerobic exercise OR moderate intensity continuous training OR resistance training OR resistance exercise OR strength training OR strength exercise OR combined training OR combined exercise OR concurrent training OR high intensity interval training OR sprint interval training OR endurance training | Postmenopause OR Post-Menopause* OR Post-menopausal Period OR Period, Post-menopausal OR Post Menopause*; Period OR Postmenopausal OR Postmenopausal Period OR old female OR elderly female OR elderly women | Vascular function OR vascular compliance OR vascular reactivity OR vascular stiffness OR vascular endothelium OR vasodilation OR endothelial function OR endothelial dysfunction OR arterial stiffness OR arterial distensibility OR pulse wave velocity OR macrovascular OR microvascular OR coronary flow reserve OR carotid intima-media thickness OR central blood pressure OR flow-mediated dilatation OR beta-stiffness index OR FMD OR PWV | Randomized controlled trial OR randomized OR placebo |
| EBSCO  [Abstract] | Exercise OR sports OR training OR aerobic training OR aerobic exercise OR moderate intensity continuous training OR resistance training OR resistance exercise OR strength training OR strength exercise OR combined training OR combined exercise OR concurrent training OR high intensity interval training OR sprint interval training OR endurance training | Postmenopause OR Post-Menopause* OR Post-menopausal Period OR Period, Post-menopausal OR Post Menopause*; Period OR Postmenopausal OR Postmenopausal Period OR old female OR elderly female OR elderly women | Vascular function OR vascular compliance OR vascular reactivity OR vascular stiffness OR vascular endothelium OR vasodilation OR endothelial function OR endothelial dysfunction OR arterial stiffness OR arterial distensibility OR pulse wave velocity OR macrovascular OR microvascular OR coronary flow reserve OR carotid intima-media thickness OR central blood pressure OR flow-mediated dilatation OR beta-stiffness index OR FMD OR PWV | Randomized controlled trial OR randomized OR placebo |

**Appendix 2.** Characteristics of the included studies.

| study | Country | Duration (wks) | Complication | Sample size | Age (mean±[SD]) | Exercise category | Summary description of exercise intervention (frequency, intensity, time excluding warm-up/cool-down and type) | supervised or nonsupervised | Outcomes |
| --- | --- | --- | --- | --- | --- | --- | --- | --- | --- |
| Tanahashi 2014 | Japan | 12 | No Complication | 10 | 61 ± 7 | CON | No exercise | supervised | IMT |
|  |  |  |  | 20 | 62 ± 6 | CET | 4.7 ± 1.2 d/wek; Initial intensity: 60% HRmax; 30min, exercise tolerance improved: 65%–80% HRmax; 40–60 min; cycling or walking |  |  |
| Prakhinkit 2014 | Thailand | 12 | NO complication | 13 | 81.0 ± 1.7 | CON | No exercise | supervised | NO, FMD |
|  |  |  |  | 13 | 74.8 ± 1.7 | CET | weeks 1–6: 3 d/wek; 20%–39% HRR; 20min, weeks 7–12: 3 d/wek; 40%–50% HRR; 30 min; traditional walking |  |  |
| Jaime 2019 | USA | 12 | NO complication | 8 | 67 ± 1.0 | CON | No exercise | supervised | AIx, PWV, FMD, |
|  |  |  |  | 12 | 64 ± 1.0 | RT | 2 d/wek; 2-3 sets*15 maximum reps at 40% 1RM; 20 min; linear periodization; low-intensity resistance exercise training |  |  |
|  |  |  |  | 13 | 64 ± 1 | HYB | 2 d/wek; 1-2 week: 24-40 Hz; 2–3 sets*15 maximum reps; 20 min, 3-12 week: 24-40 Hz; 2–3 sets*15 maximum reps; 30-35 min; whole-body vibration training |  |  |
| He 2022 | China | 8 | NO complication | 15 | 58.33 ± 3.06 | CON | No exercise | supervised | NO, FMD |
|  |  | 8 |  | 15 | 54.3 ± 6.4 | CET | 3 d/wek; 70%–80% HRmax; 40 min; treadmill training; |  |  |
|  |  | 8 |  | 10 | 55.8 ± 5.6 | INT | 3 d/wek; 4 min at 75%-85% HRmax; active rest intervals: 3 min at 50-60% HRmax; treadmill training; HIIT |  |  |
| Ho 2020 | Australia | 8 | NO complication | 30 | 53.9 ± 3.39 | CON | No exercise | supervised | AIx, PWV |
|  |  |  |  | 30 | 53.2 ± 3.07 | INT | 3 d/wek; work intervals: 8-sec sprints at near-maximal exertion; 120 RPM; active recovery intervals: 12-sec at 50‐60 RPM; 20 min; cycling |  |  |
| Teixeira 2019 | Brazil | 16 | NO complication | 10 | 55.8 ± 3.9 | CON | slight stretching | supervised | NO |
|  |  |  |  | 10 | 56 ± 4.5 | RT | 3 d/wek; 60 min; 8 exercises; 1-3 weeks: 30-50% 1RM; 4-15 weeks: 60-75% 1RM; resistance exercise |  |  |
| Figueroa 2011 | Korea | 12 | NO complication | 12 | 54 ± 1 | CON | No exercise | supervised | PWV |
|  |  |  |  | 12 | 54 ± 2 | CT | 3 d/wek, RT (2 sets*12 reps; 60% 1RM; 15 sec rest per set; 20 min; 9 exercises) and CET (60% HRmax, 20 min, running) |  |  |
| Figueroa A  2014 | USA | 6 | hypertension | 13 | 56 ± 3 | CON | No exercise | supervised | AIx |
|  |  |  |  | 15 | 56 ± 3 | HYB | 3 d/wek; 1-2 sets*30-60 sec reps; 60 sec rest per set; lower limbs training (4 exercises) at 25–35 Hz and amplitude 1 mm; WBV training |  |  |
| Figueroa A  2013 | USA | 12 | hypertension | 12 | 56.4 ± 1 | CON | No exercise | supervised | PWV |
|  |  |  |  | 13 | 55.5 ± 0.7 | HYB | 3 d/wek; 1-6 sets*30-60 sec reps; 60-30 sec rest per set; lower limbs training (4 exercises) at 25–35 Hz and amplitude 1 mm; 30-60 sec; linear periodized; WBV training |  |  |
| Ha 2018 | Korea | 12 | NO complication | 8 | 76.00 ± 5.52 | CON | No exercise | nonsupervised | AIx, PWV |
|  |  |  |  | 11 | 74.09 ± 4.21 | CET | 3 d/wek; 30-60% HRR; 40-min; Weeks 1–4: RPE 9–10; Weeks 4–8: RPE 11–12; Weeks 9–12: RPE 13–14; running |  |  |
| Ohta 2012 | Japan | 12 | NO complication | 13 | 71.5 ± 7.4 | CON | No exercise | nonsupervised | PWV |
|  |  |  |  | 13 | 72.2 ± 4.2 | CET | 3 d/wek, 10-20 min, 40 steps/min at lactate threshold, 60%-70% HRmax, bench step exercise |  |  |
| Casey 2007 | USA | 18 | NO complication | 13 | 58.7 ± 4.5 | RT | 2 d/wek; 1 set*12 reps at 50 % 1RM; whole-body (10 exercises); 30 min; linear periodized; resistance training | supervised | FMD |
|  |  |  |  | 10 | 59.7 ± 6.5 | CET | 2 d/wek; 30-40 min; 65-80% HRR; treadmill walking; aerobic training |  |  |
| Wong  2018 | Korea | 12 | stage-2 hypertension | 21 | 59 ± 1 | CON | No exercise | supervised | PWV |
|  |  |  |  | 20 | 59 ± 1 | INT | 2-5 d/wek; 3 sets*64 steps; 3 work intervals* 6 min at 11-13 RPE; 2 active rest intervals*10 min; linear periodized; stair climbing |  |  |
| Jo 2020 | Korea | 12 | NO complication | 21 | 62.5 ± 13.9 | CON | No exercise | supervised | FMD |
|  |  |  |  | 22 | 57.3 ± 8.4 | HYB | 3 d/wek; 40-80% HRR; 40 min; exergame |  |  |
|  |  |  |  | 22 | 61.8 ± 10.1 | CET | 3 d/wek; 40 min; 60-80% HRR; walking or jogging on treadmill |  |  |
| N`eve 2023 | Finland | 18 | NO complication | 87 | 66.6 ± 5.4 | CON | No exercise | supervised | IMT |
|  |  |  |  | 101 | 66.9 ± 5.2 | CET | 3-5 d/wek; 30-60 min; 40%-60% VO_2_max; skiing, walking, cycling |  |  |
|  |  |  |  | 90 | 66.5 ± 5.3 | RT | 2 d/wek; 2 sets*15 reps at 60% 1RM; whole-body exercise |  |  |
| Lee 2019 | Korea | 12 | stage-2 hypertension | 10 | 70 ± 4 | CON | slight stretching | supervised | PWV |
|  |  |  |  | 10 | 71 ± 4 | HYB | 3 d/wek; 60 min; 30-60% HRR; taekwondo training |  |  |
| Jung 2022 | Korea | 12 | Obesity | 14 | 74.64 ± 5.77 | CON | No exercise | supervised | PWV |
|  |  |  |  | 14 | 75.36 ± 4.50 | CET | 3 d/wek; 1-2 weeks: 2 set*1 rep; 25 minute, 3-8 weeks: 3 set*1 rep; 40 minute; 9-12 weeks: 4 set*1 rep; 55 minute; HRR 60-80%; 10 exercises; circuit training |  |  |
| Park J  2017 | Korea | 24 | Obesity | 20 | 68.4 ± 2.6 | CON | No exercise | supervised | IMT |
|  |  |  |  | 21 | 70.4 ± 4.5 | CT | 3-5 d/wk; RT (1-12 weeks: 2-3 sets*8-12 reps, 13-24 weeks: 2-3 sets*13-15 reps; 1-2 rest intervals*1-2 min; band exercise; 10 exercises; linear periodization; 20-30 min) and CET (RPE: 5-6, 40-50 min, walking) |  |  |
| Fetter 2020 | Brazil | 12 | hypertension | 14 | 59 ± 1.1 | CON | slight stretching | supervised | PWV, FMD, AIx |
|  |  |  |  | 9 | 58.00 ± 1.2 | HYB | 2 d/wek; 60 min; three full sequences of sun salutations; yoga and respiratory |  |  |
| Miura 2015 | Japan | 12 | hypertension | 53 | 71.8 ± 5.6 | CON | No exercise | supervised | PWV |
|  |  |  |  | 55 | 72.0 ± 7.1 | RT | 2 d/wek; 3-5 sets*15-20 reps; 40 min; 6-8 exercises; linear periodization; circuit training; rubber tube and/or lightweight dumbbells |  |  |
| Park Jinkee  2017 | Korea | 24 | Obesity | 25 | 74.7 ± 5.1 | CON | No exercise | supervised | IMT |
|  |  |  |  | 25 | 73.5 ± 7.1 | CT | 3-5 d/wek; RT (1-12 weeks: 2-3sets*8-11 reps; 13-24 weeks: 2-3 sets*12-15 reps; 20-30 min; 1-2 rest interval*1-2 min; elastic band exercise; 12 exercises) and CET (30-50 min; RPE:13-17; walking) |  |  |
| Son 2016 | Korea | 12 | hypertension | 10 | 74.7 ± 2 | CON | No exercise | supervised | PWV, NO |
|  |  |  |  | 10 | 76 ± 5 | CT | 3 d/wek; RT (1-4 weeks: 40-50% HRR; 5-8 weeks: 50-60% HRR, 9-12 weeks: 60-70% HRR; 40 min; 10 exercises; resistant band exercise) and CET (1-4 weeks: 40-50% HRR, 9-12 weeks: 60-70% HRR; 30 min; walking) |  |  |
| Taha 2016 | Egypt | 10 | hypertension | 23 | 47.78 ± 2.59 | CON | No exercise | supervised | NO |
|  |  |  |  | 23 | 48.17 ± 2.20 | INT | 3 d/wek; 40 min; 4 work intervals: 4 min; 80-85% HRR; 3 active rest intervals: 3 min; 70% HRR; walking/running |  |  |
| Swift 2012 | USA | 24 | hypertension | 23 | 56.8 ± 5.4 | CON | No exercise | supervised | FMD |
|  |  |  |  | 68 | 57.4 ± 5.8 | CET | 3-4 d/wek; 50% VO_2_peak; 30 min; cycling/running |  |  |
| Figueroa 2013 | USA | 12 | Obesity | 13 | 54 ± 1 | CON | slight stretching | supervised | PWV |
|  |  |  |  | 14 | 54 ± 1 | RT | 3 d/wek; 1-2 weeks: 2 sets*18-22 reps; 3-12 weeks: 3 sets*18-22 reps; low-intensity resistance exercise training |  |  |
| Seals 2001 | USA | 13 | NO complication | 18 | 62 ± 9 | CON | No exercise | nonsupervised | PWV |
|  |  |  |  | 17 | 62 ± 9 | CET | Phase 1: 3-4 d/wek; 30 min; 40-50% HRmax, Phase 2: >3-4 d/wek; 40-45 min; 65-80% HRmax; walking |  |  |
| Park 2023 | Korea | 8 | NO complication | 15 | 82 ± 4.6 | CON | No exercise | supervised | PWV, FMD |
|  |  |  |  | 15 | 82.9 ± 2.7 | CET | 3 d/wek; 50-60% HRmax; 45 min; walking; |  |  |
| Kujawski 2018 | Poland | 12 | NO complication | 27 | 64 ± 1 | RT | 2 d/wek; 6 exercises; 4 sets*10-15 reps; 50 min; linear periodized RT; resistance exercise | supervised | PWV |
|  |  |  |  | 28 | 66 ± 1 | HYB | 2 d/wek; 15-20 exercises; 40%-50% HRR; 45 min; sitting calisthenics balance |  |  |
| Shin 2015 | Korea | 12 | NO complication | 14 | 62.7 ± 5.9 | CON | No exercise | nonsupervised | FMD, PWV, IMT |
|  |  |  |  | 29 | 64.0 ± 5.4 | HYB | 1 d/wek; 60 min; Tai Chi |  |  |
| Yoshizawa 2010 | Japan | 8 | NO complication | 10 | 58 ± 1 | CON | No exercise | supervised | FMD |
|  |  |  |  | 10 | 57 ± 1 | CET | 3-5 d/wek; 25-45 min; 60-75% HRmax; walking or cycling |  |  |
| Figueroa Arturo 2013 | USA | 12 | NO complication | 13 | 54 ± 1 | CON | No exercise | supervised | PWV |
|  |  |  |  | 14 | 54 ± 1 | RT | 3 d/wek; 4 exercises; 2 sets*18-22 reps; 30 min; linear periodized RT; resistance exercise |  |  |
| Turky 2013 | Egypt | 8 | Hypertension | 13 | 52.9 ± 2.6 | CON | No exercise | supervised | NO |
|  |  |  |  | 12 | 52.7 ± 2.2 | CET | 3 d/wek; 20 min; 60-70% HRmax; walking |  |  |

Abbreviations: CON, control; CET, continuous endurance training; INT, interval training; RT, resistance training; CT, combined training; HYB, hybrid-type training; MIIT, moderate-intensity interval training; RM, repetition maximum; HIIT, high-intensity interval training; MICT, moderate intensity continuous training; SIT, sprint interval training; WBV training, whole-body vibration training; VO_2_max, maximal oxygen uptake; VO_2_peak, peak oxygen uptake; reps, repetitions; HRmax, maximum heart rate; HRR, heart rate reserve; FMD, flow mediated dilation, PWV, pulse wave velocity, AIx, Augmentation Index, IMT, intima-media thickness, NO, Nitric oxide.

**Appendix 3.** List of included studies.

1. Tanahashi, K., Akazawa, N., Miyaki, A., Choi, Y., Ra, S. G., Matsubara, T., . . . Maeda, S. (2014). Aerobic exercise training decreases plasma asymmetric dimethylarginine concentrations with increase in arterial compliance in postmenopausal women. *Am J Hypertens, 27*(3), 415-421. doi:10.1093/ajh/hpt217.
2. Prakhinkit, S., Suppapitiporn, S., Tanaka, H., & Suksom, D. (2014). Effects of Buddhism walking meditation on depression, functional fitness, and endothelium-dependent vasodilation in depressed elderly. *J Altern Complement Med, 20*(5), 411-416. doi:10.1089/acm.2013.0205.
3. Jaime, S. J., Maharaj, A., Alvarez-Alvarado, S., & Figueroa, A. (2019). Impact of low-intensity resistance and whole-body vibration training on aortic hemodynamics and vascular function in postmenopausal women. *Hypertens Res, 42*(12), 1979-1988. doi:10.1038/s41440-019-0328-1. doi:10.1038/s41440-019-0328-1.
4. He, H., Wang, C., Chen, X., Sun, X., Wang, Y., Yang, J., & Wang, F. (2022). The effects of HIIT compared to MICT on endothelial function and hemodynamics in postmenopausal females. *J Sci Med Sport, 25*(5), 364-371. doi:10.1016/j.jsams.2022.01.007. doi:10.1016/j. jsams.2022.01.007.
5. Ho, T. Y., Redmayne, G. P., Tran, A., Liu, D., Butlin, M., Avolio, A., . . . Boutcher, Y. N. (2020). The effect of interval sprinting exercise on vascular function and aerobic fitness of post-menopausal women. *Scand J Med Sci Sports, 30*(2), 312-321. doi:10.1111/sms.13574. doi:10.1111/sms.13574.
6. Teixeira, B. C., Boeno, F. P., Siqueira, C. V., Cadore, E. L., Ribeiro, J. L., Reischak-Oliveira, A., & dos Santos Cunha, G. (2019). Strength training enhances endothelial and muscular function in postmenopausal women. *Science & sports, 34*(2), e147-e154. doi: https://doi.org/10.1016/j.scispo.2018.09.006.
7. Figueroa, A., Park, S. Y., Seo, D. Y., Sanchez-Gonzalez, M. A., & Baek, Y. H. (2011). Combined resistance and endurance exercise training improves arterial stiffness, blood pressure, and muscle strength in postmenopausal women. *Menopause, 18*(9), 980-984. doi:10.1097/gme.0b013e3182135442. doi:10.1097/gme.0b013e3182135442
8. Figueroa, A., Kalfon, R., Madzima, T. A., & Wong, A. (2014). Effects of whole-body vibration exercise training on aortic wave reflection and muscle strength in postmenopausal women with prehypertension and hypertension. *J Hum Hypertens, 28*(2), 118-122. doi:10.1038/jhh.2013.59
9. Figueroa, A., Kalfon, R., Madzima, T. A., & Wong, A. (2014). Whole-body vibration exercise training reduces arterial stiffness in postmenopausal women with prehypertension and hypertension. *Menopause, 21*(2), 131-136. doi:10.1097/GME.0b013e318294528c
10. Ha, M. S., Kim, J. H., Kim, Y. S., & Kim, D. Y. (2018). Effects of aquarobic exercise and burdock intake on serum blood lipids and vascular elasticity in Korean elderly women. *Exp Gerontol, 101*, 63-68. doi:10.1016/j.exger.2017.11.005
11. Ohta, M., Hirao, N., Mori, Y., Takigami, C., Eguchi, M., Tanaka, H., . . . Yamato, H. (2012). Effects of bench step exercise on arterial stiffness in post-menopausal women: contribution of IGF-1 bioactivity and nitric oxide production. *Growth Horm IGF Res, 22*(1), 36-41. doi:10.1016/j.ghir.2011.12.004
12. Casey, D. P., Pierce, G. L., Howe, K. S., Mering, M. C., & Braith, R. W. (2007). Effect of resistance training on arterial wave reflection and brachial artery reactivity in normotensive postmenopausal women. *Eur J Appl Physiol, 100*(4), 403-408. doi:10.1007/s00421-007-0447-2
13. Wong, A., Figueroa, A., Son, W. M., Chernykh, O., & Park, S. Y. (2018). The effects of stair climbing on arterial stiffness, blood pressure, and leg strength in postmenopausal women with stage 2 hypertension. *Menopause, 25*(7), 731-737. doi:10.1097/gme.0000000000001072
14. Jo, E. A., Wu, S. S., Han, H. R., Park, J. J., Park, S., & Cho, K. I. (2020). Effects of exergaming in postmenopausal women with high cardiovascular risk: A randomized controlled trial. *Clin Cardiol, 43*(4), 363-370. doi:10.1002/clc.23324
15. Nève, G., Komulainen, P., Savonen, K., Hassinen, M., Männikkö, R., Infanger, D., . . . Rauramaa, R. (2023). Effect of lifestyle interventions on carotid arterial structure - The DR's EXTRA study. *Prev Med, 168*, 107436. doi:10.1016/j.ypmed.2023.107436
16. Lee, S. H., Scott, S. D., Pekas, E. J., Lee, S., Lee, S. H., & Park, S. Y. (2019). Taekwondo training reduces blood catecholamine levels and arterial stiffness in postmenopausal women with stage-2 hypertension: randomized clinical trial. *Clin Exp Hypertens, 41*(7), 675-681. doi:10.1080/10641963.2018.1539093
17. Jung, W.-S., Kim, Y.-Y., Kim, J.-W., & Park, H.-Y. (2022). Effects of Circuit Training Program on Cardiovascular Risk Factors, Vascular Inflammatory Markers, and Insulin-like Growth Factor-1 in Elderly Obese Women with Sarcopenia. *RCM, 23*(4). Retrieved from {<https://www.imrpress.com/RCM/articles/10.31083/j.rcm2304134>}. doi:10.31083/j.rcm2304134
18. Park, J., & Park, H. (2017). Effects of 6 months of aerobic and resistance exercise training on carotid artery intima media thickness in overweight and obese older women. *Geriatr Gerontol Int, 17*(12), 2304-2310. doi:10.1111/ggi.12972
19. Fetter, C., Marques, J. R., de Souza, L. A., Dartora, D. R., Eibel, B., Boll, L. F. C., . . . Irigoyen, M. C. (2020). Additional Improvement of Respiratory Technique on Vascular Function in Hypertensive Postmenopausal Women Following Yoga or Stretching Video Classes: The YOGINI Study. *Front Physiol, 11*, 898. doi:10.3389/fphys.2020.00898
20. Miura, H., Takahashi, Y., Maki, Y., & Sugino, M. (2015). Effects of exercise training on arterial stiffness in older hypertensive females. *Eur J Appl Physiol, 115*(9), 1847-1854. doi:10.1007/s00421-015-3168-y
21. Park, J., Kwon, Y., & Park, H. (2017). Effects of 24-Week Aerobic and Resistance Training on Carotid Artery Intima-Media Thickness and Flow Velocity in Elderly Women with Sarcopenic Obesity. *Journal of atherosclerosis and thrombosis, 24*(11), 1117-1124. doi:10.5551/jat.39065
22. Son, W. M., Sung, K. D., Cho, J. M., & Park, S. Y. (2017). Combined exercise reduces arterial stiffness, blood pressure, and blood markers for cardiovascular risk in postmenopausal women with hypertension. *Menopause, 24*(3), 262-268. doi:10.1097/gme.0000000000000765
23. Taha, M. (2016). Effect of High Intensity Interval Training on Endothelial Function in Postmenopausal Hypertensive Patients:Randomized Controlled Trial. *International journal of physiotherapy, 3*. doi:10.15621/ijphy/2016/v3i1/88908
24. Swift, D. L., Earnest, C. P., Blair, S. N., & Church, T. S. (2012). The effect of different doses of aerobic exercise training on endothelial function in postmenopausal women with elevated blood pressure: results from the DREW study. *Br J Sports Med, 46*(10), 753-758. doi:10.1136/bjsports-2011-090025
25. Figueroa, A., Vicil, F., Sanchez-Gonzalez, M. A., Wong, A., Ormsbee, M. J., Hooshmand, S., & Daggy, B. (2013). Effects of Diet and/or Low-Intensity Resistance Exercise Training on Arterial Stiffness, Adiposity, and Lean Mass in Obese Postmenopausal Women. *American journal of hypertension, 26*(3), 416-423. Retrieved from <https://doi.org/10.1093/ajh/hps050>. doi:10.1093/ajh/hps050
26. Seals, D. R., Tanaka, H., Clevenger, C. M., Monahan, K. D., Reiling, M. J., Hiatt, W. R., . . . DeSouza, C. A. (2001). Blood pressure reductions with exercise and sodium restriction in postmenopausal women with elevated systolic pressure: role of arterial stiffness. *J Am Coll Cardiol, 38*(2), 506-513. doi:10.1016/s0735-1097(01)01348-1
27. Park, W., Lee, J., Hong, K., Park, H. Y., Park, S., Kim, N., & Park, J. (2023). Protein-Added Healthy Lunch-Boxes Combined with Exercise for Improving Physical Fitness and Vascular Function in Pre-Frail Older Women: A Community-Based Randomized Controlled Trial. *Clin Interv Aging, 18*, 13-27. doi:10.2147/cia.S391700
28. Kujawski, S., Kujawska, A., Gajos, M., Klawe, J. J., Tafil-Klawe, M., Mądra-Gackowska, K., . . . Zalewski, P. (2018). Effects of 3-months sitting callisthenic balance and resistance exercise on aerobic capacity, aortic stiffness and body composition in healthy older participants. Randomized Controlled Trial. *Exp Gerontol, 108*, 125-130. doi:10.1016/j.exger.2018.04.009
29. Shin, J. H., Lee, Y., Kim, S. G., Choi, B. Y., Lee, H. S., & Bang, S. Y. (2015). The beneficial effects of Tai Chi exercise on endothelial function and arterial stiffness in elderly women with rheumatoid arthritis. *Arthritis Res Ther, 17*, 380. doi:10.1186/s13075-015-0893-x
30. Yoshizawa, M., Maeda, S., Miyaki, A., Misono, M., Choi, Y., Shimojo, N., . . . Tanaka, H. (2010). Additive beneficial effects of lactotripeptides intake with regular exercise on endothelium-dependent dilatation in postmenopausal women. *Am J Hypertens, 23*(4), 368-372. doi:10.1038/ajh.2009.270
31. Figueroa, A., Vicil, F., Sanchez-Gonzalez, M. A., Wong, A., Ormsbee, M. J., Hooshmand, S., & Daggy, B. (2013). Effects of diet and/or low-intensity resistance exercise training on arterial stiffness, adiposity, and lean mass in obese postmenopausal women. *Am J Hypertens, 26*(3), 416-423. doi:10.1093/ajh/hps050
32. Khalid, T., Nesreen, E., & Ramadhan, O. (2013). Effects of exercise training on postmenopausal hypertension: implications on nitric oxide levels. *Med J Malaysia, 68*(6), 459-464.

**Appendix 4.** Risk of bias assessment.


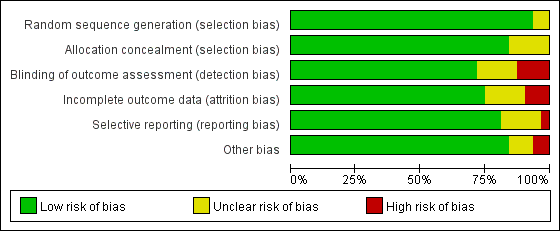

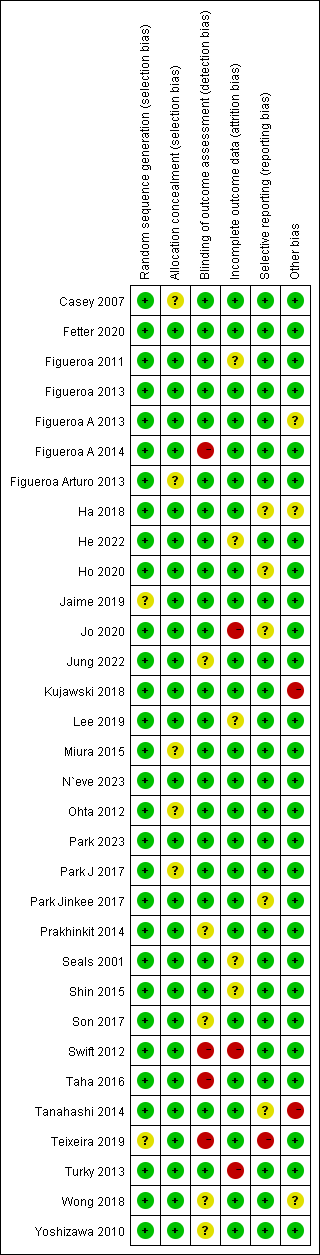


**Appendix 5.** Contributions of direct and indirect comparisons to NMA and the number of studies of each direct comparison of flow mediated dilation (FMD), pulse wave velocity (PWV), augmentation index (AIx), intima-media thickness (IMT), nitric oxide (NO).


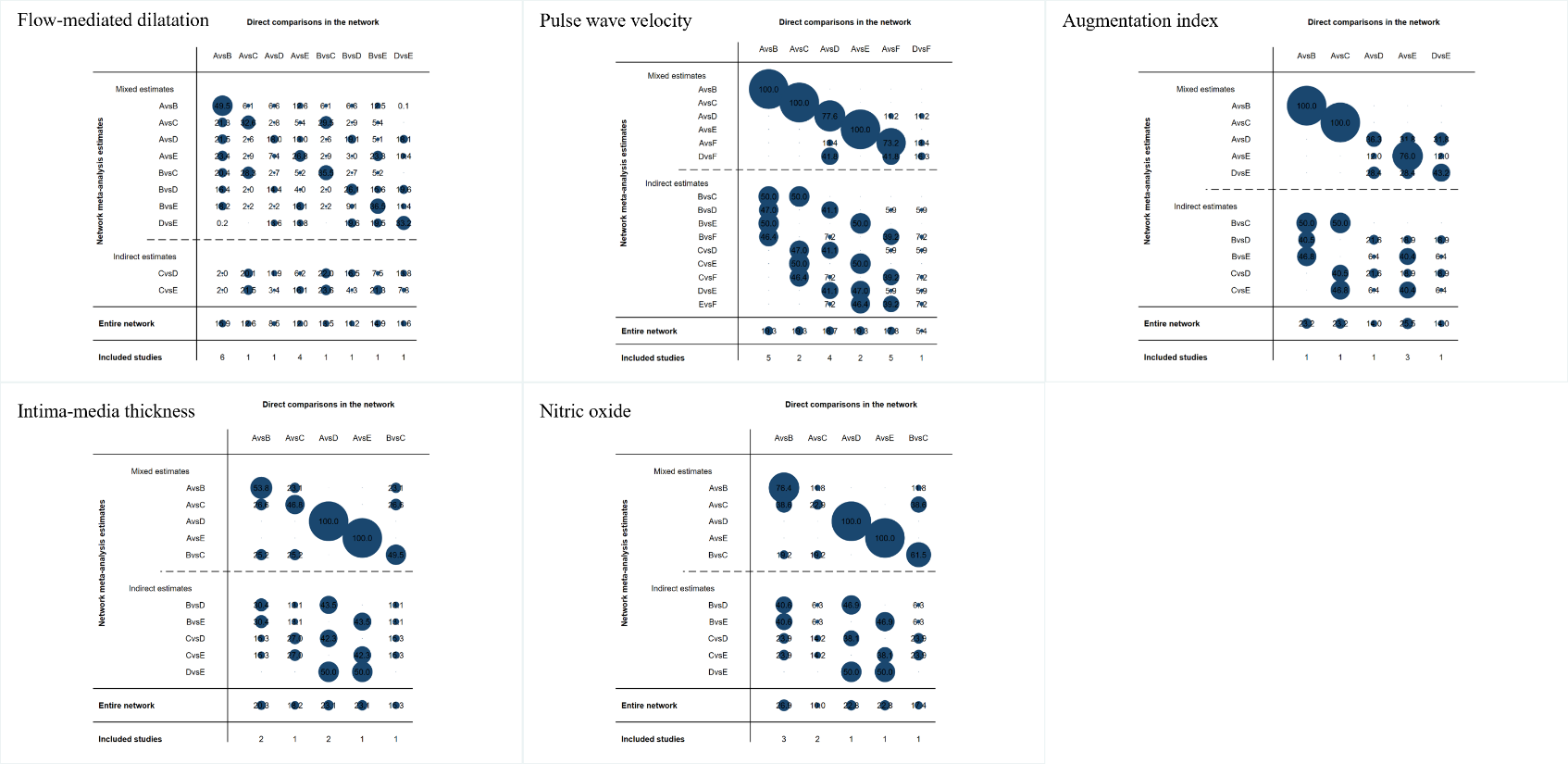


**Appendix 6.** Inconsistency of vascular function outcomes tested by loop-specific heterogeneity estimates, inconsistency model, and node splitting analysis.

| **loop-specific heterogeneity estimates** | | | | | | |
| --- | --- | --- | --- | --- | --- | --- |
| **Loop** | **IF** | **seIF** | **z_value** | **p_value** | **CI_95** | **Loop_Hete rog_tau2** |
| **Flow mediated dilation** | | | | | | |
| CET-RT-HYB | 0.94 | 0.66 | 1.42 | 0.16 | (0.00,2.22) | 0.00 |
| CON-CET-RT | 0.86 | 0.96 | 0.90 | 0.37 | (0.00,2.73) | 0.21 |
| CON-CET-INT | 0.81 | 0.90 | 0.90 | 0.37 | (0.00,2.58) | 0.14 |
| CON-CET-HYB | 0.69 | 0.79 | 0.87 | 0.38 | (0.00,2.23) | 0.32 |
| CON-RT-HYB | 0.62 | 0.68 | 0.92 | 0.36 | (0.00,1.95) | 0.00 |
| **Pulse wave velocity** | | | | | | |
| CON-RT-HYB | 0.22 | 0.47 | 0.47 | 0.64 | (0.00,1.15) | 0.00 |
| **Augmentation index** | | | | | | |
| CON-RT-HYB | 0.29 | 0.69 | 0.42 | 0.67 | (0.00,1.64) | 0.00 |
| **Intima-media thickness** | | | | | | |
| CON-CET-RT | 0.06 | 0.44 | 0.13 | 0.90 | (0.00,0.92) | 0.00 |
| **Nitric oxide** | | | | | | |
| CON-CET-INT | 0.03 | 0.88 | 0.04 | 0.97 | (0.00,1.75) | 0.00 |

Abbreviations: CON, control; CET, continuous endurance training; INT, interval training; RT, resistance training; CT, combined training; HYB, hybrid-type training; MIIT, moderate-intensity interval training; RM, repetition maximum; HIIT, high-intensity interval training; MICT, moderate intensity continuous training; SIT, sprint interval training; WBV training, whole-body vibration training;

| **Inconsistency model** | | | | | |
| --- | --- | --- | --- | --- | --- |
|  | **Flow mediated dilation** | **Pulse wave velocity** | **Augmentation index** | **Intima-media thickness** | **Nitric oxide** |
| chi2 | 4.97 | 1.28 | 0.24 | 0.02 | 1.98 |
| Prob > chi2 | 0.42 | 0.53 | 0.63 | 0.89 | 0.37 |

| **Node splitting analysis** | | | | | | | |
| --- | --- | --- | --- | --- | --- | --- | --- |
| **Side** | **Direct** |  | **Indirect** |  | **Difference** |  |  |
|  | **Coef.** | **Std. Err.** | **Coef.** | **Std. Err.** | **Coef.** | **Std. Err.** | **P>z** |
| **Flow mediated dilation** | | | | | | | |
| A B * | 0.92 | 0.27 | 0.81 | 0.82 | 0.11 | 0.87 | 0.90 |
| A C * | 2.32 | 0.69 | 3.86 | 1.20 | (1.54) | 1.31 | 0.24 |
| A D | 1.64 | 0.71 | 0.66 | 0.63 | 0.98 | 0.97 | 0.31 |
| A E * | 0.96 | 0.33 | 1.44 | 1.09 | (0.49) | 1.14 | 0.67 |
| B C * | 2.06 | 0.68 | 0.52 | 1.22 | 1.54 | 1.31 | 0.24 |
| B D | (0.14) | 0.67 | 0.48 | 0.66 | (0.62) | 0.94 | 0.51 |
| B E | 0.67 | 0.56 | (0.23) | 0.42 | 0.91 | 0.69 | 0.19 |
| D E | (0.11) | 0.67 | (0.07) | 0.76 | (0.04) | 1.01 | 0.97 |
| **Note:** A: CON, B: CET, C: INT, D: RT, E: HYB. | | | | | | | |
| **Pulse wave velocity** | | | | | | | |
| A D * | (0.36) | 0.20 | (1.17) | 0.89 | 0.81 | 0.92 | 0.38 |
| A F * | (0.67) | 0.20 | (0.81) | 0.88 | 0.14 | 0.91 | 0.88 |
| D F | (0.13) | 0.44 | (0.35) | 0.31 | 0.22 | 0.54 | 0.68 |
| **Note:** A: CON, B: CET, C: INT, D: RT, E: CT, F: HYB. | | | | | | | |
|  | | | | | | | |
| **Augmentation index** | | | | | | | |
| A D * | 0.29 | 0.51 | 0.89 | 1.08 | (0.60) | 1.22 | 0.63 |
| D E * | (1.01) | 0.48 | (0.41) | 1.12 | (0.60) | 1.22 | 0.63 |
| **Note:** A: CON, B: CET, C: INT, D: RT, E: HYB. | | | | | | | |
|  | | | | | | | |
| **Intima-media thickness** | | | | | | | |
| A C * | 0.07 | 0.15 | 0.19 | 0.81 | (0.12) | 0.83 | 0.89 |
| B C * | 0.22 | 0.15 | 0.11 | 0.82 | 0.12 | 0.83 | 0.89 |
| **Note:** A: CON, B: CET, C: RT, D: CT, E: HYB. | | | | | | | |
|  | | | | | | | |
| **Nitric oxide** | | | | | | | |
| A B * | 1.52 | 0.48 | 4.79 | 2.72 | (3.27) | 2.77 | 0.24 |
| A C * | 5.70 | 0.86 | 6.84 | 2.39 | (1.14) | 2.49 | 0.65 |
| B C | 3.99 | 1.19 | 4.36 | 1.47 | (0.38) | 1.87 | 0.84 |
| **Note:** A: CON, B: CET, C: INT, D: RT, E: CT. | | | | | | | |

* All the evidence about these contrasts comes from the trials which directly compare them.

**Appendix 7.** Forest plots of eligible comparisons of flow mediated dilation (FMD), pulse wave velocity (PWV), augmentation index (AIx), intima-media thickness (IMT), nitric oxide (NO).


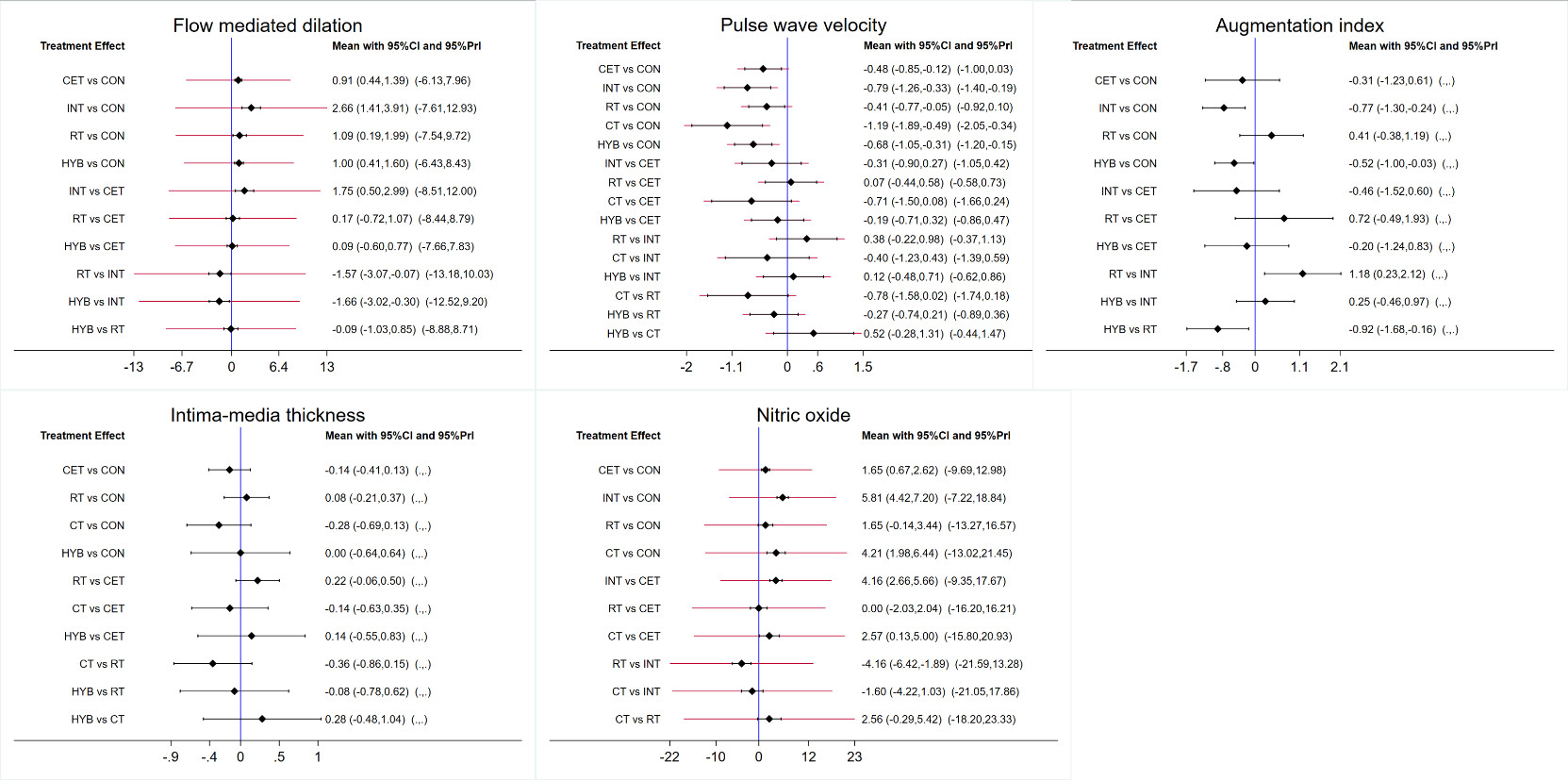


Abbreviations: CON, control, CET, continuous endurance training; CT, combined training; HYB, hybrid-type training; INT, interval training; RT, resistance training.

**Appendix 8.** The funnel plot graphics of flow mediated dilation (FMD), pulse wave velocity (PWV), augmentation index (AIx), intima-media thickness (IMT), nitric oxide (NO) in NMA.


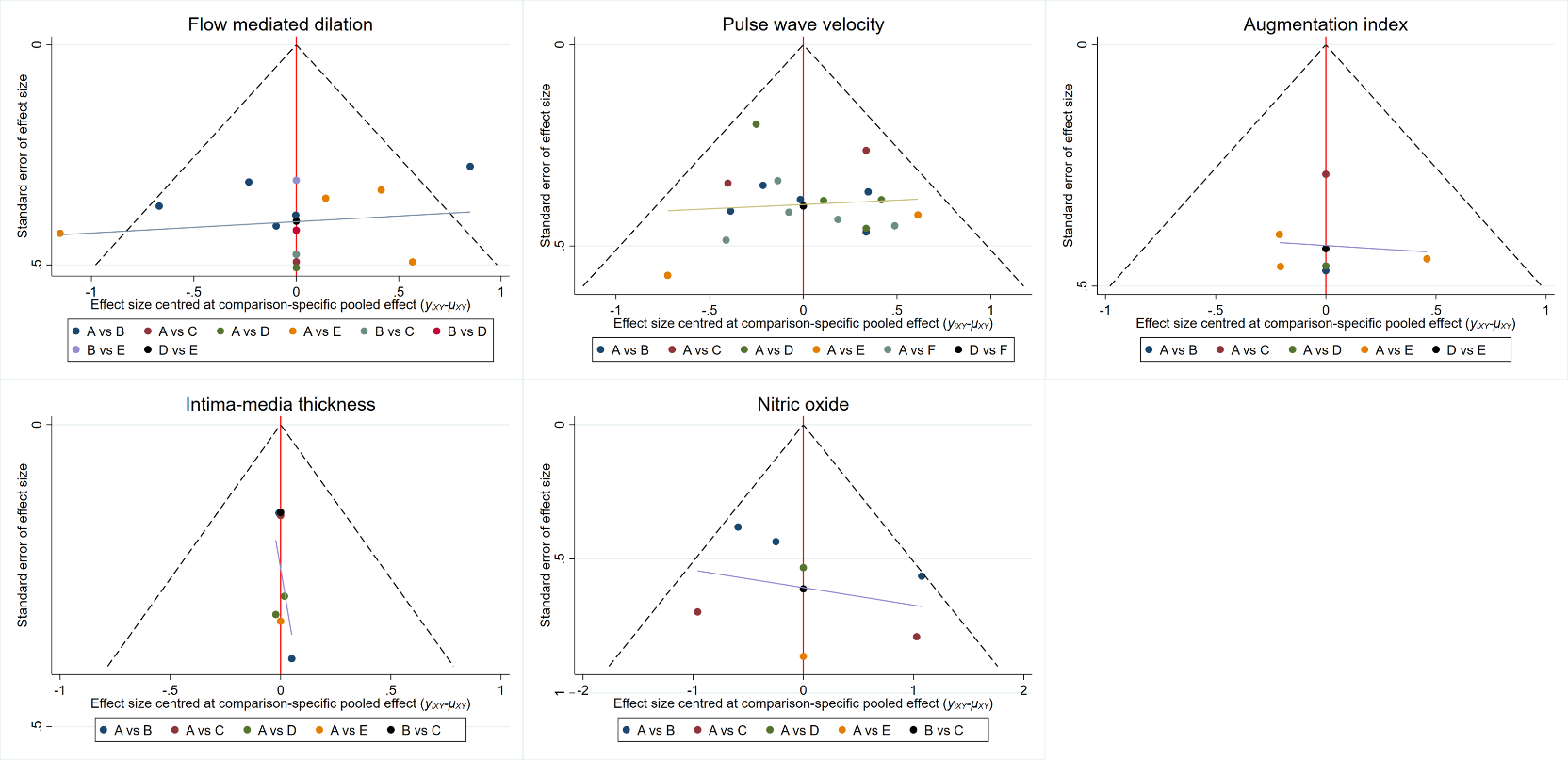


**Appendix 9.** Area under the curve for cumulative ranking probability of each intervention on flow mediated dilation (FMD), pulse wave velocity (PWV), augmentation index (AIx), intima-media thickness (IMT), nitric oxide (NO).


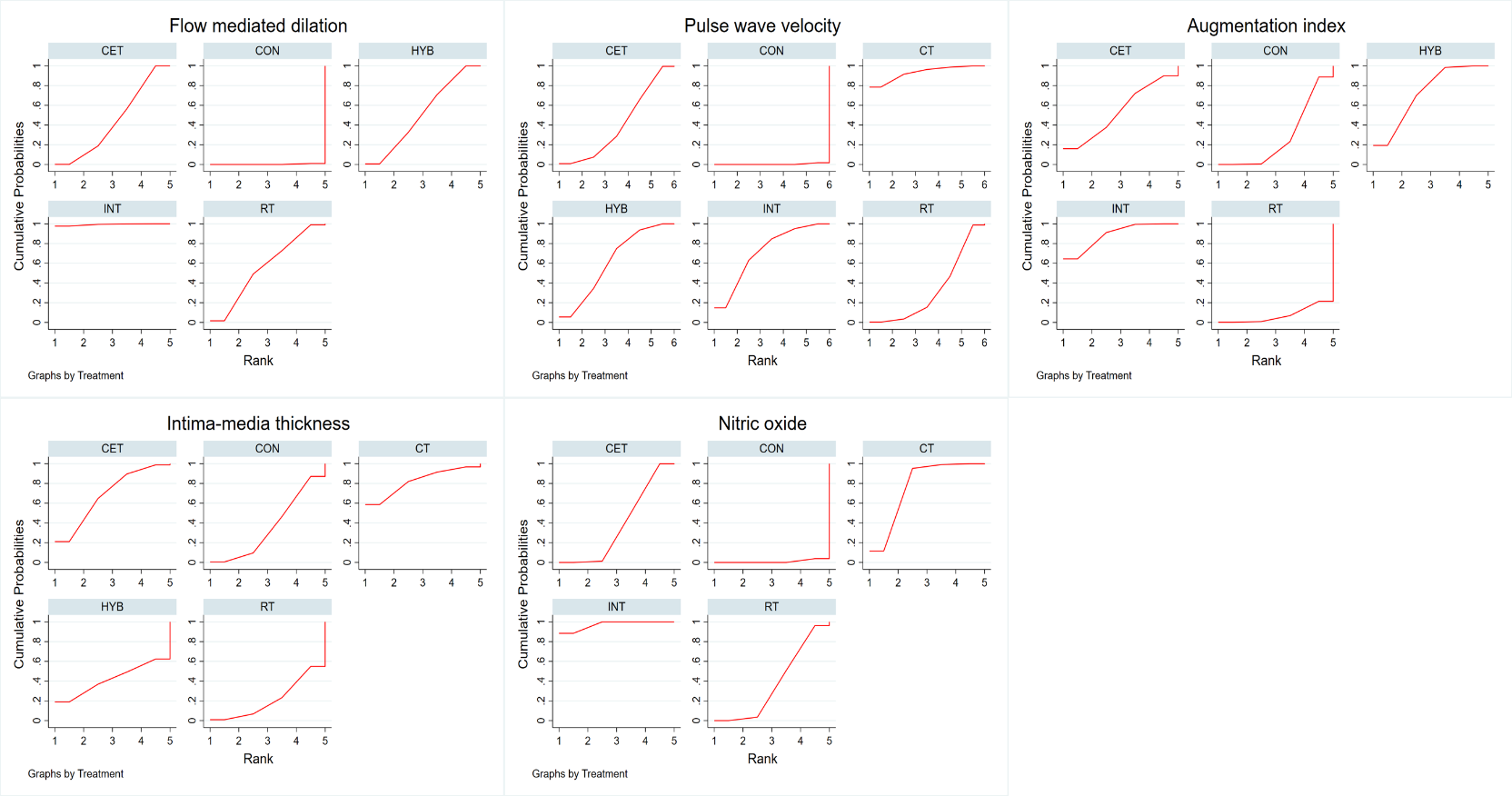


Abbreviations: CON, control, CET, continuous endurance training; CT, combined training; HYB, hybrid-type training; INT, interval training; RT, resistance training.
